# Effects of phytoplankton, viral communities, and warming on free-living and particle-associated marine prokaryotic community structure

**DOI:** 10.1038/s41467-022-35551-4

**Published:** 2022-12-23

**Authors:** Yi-Chun Yeh, Jed A. Fuhrman

**Affiliations:** Department of Biological Sciences, University of Southern California, Los Angeles, CA 90089-0371 USA

**Keywords:** Microbial ecology, Microbial ecology, Marine biology

## Abstract

Free-living and particle-associated marine prokaryotes have physiological, genomic, and phylogenetic differences, yet factors influencing their temporal dynamics remain poorly constrained. In this study, we quantify the entire microbial community composition monthly over several years, including viruses, prokaryotes, phytoplankton, and total protists, from the San-Pedro Ocean Time-series using ribosomal RNA sequencing and viral metagenomics. Canonical analyses show that in addition to physicochemical factors, the double-stranded DNA viral community is the strongest factor predicting free-living prokaryotes, explaining 28% of variability, whereas the phytoplankton (via chloroplast 16S rRNA) community is strongest with particle-associated prokaryotes, explaining 31% of variability. Unexpectedly, protist community explains little variability. Our findings suggest that biotic interactions are significant determinants of the temporal dynamics of prokaryotes, and the relative importance of specific interactions varies depending on lifestyles. Also, warming influenced the prokaryotic community, which largely remained oligotrophic summer-like throughout 2014–15, with cyanobacterial populations shifting from cold-water ecotypes to warm-water ecotypes.

## Abstract (web summary)

Over several years, this study examines how biotic interactions and warming affect the entire marine prokaryotic community at a location off the coast of Southern California. Analyses show that free-living and particle-associated prokaryotes were strongly predicted by phytoplankton and viral communities, and El Niño warming shifted cyanobacteria from cold-water ecotypes to warm-water ecotypes.

## Introduction

Planktonic prokaryotes dominate the oceanic biogeochemical cycling^1–3^, thus understanding how prokaryotic communities are structured, and identifying the underlying processes, are crucial to predict their responses to environmental changes. Generally, community dynamics of prokaryotes are driven by a combination of environmental variables, such as temperature and nutrients availability^4–9^, plus biotic interactions (e.g., predation, parasitism, mutualism, or competition) with other organisms, including phytoplankton, protists, and viruses^10–16^. However, studies taking all these components into account remain limited.

In aquatic ecosystems, prokaryotes can live either freely (as unattached individuals) or attached to particles, and these two communities have physiological, genomic, and phylogenetic differences^17–21^. Free-living prokaryotes, dominated by groups such as SAR11, often have streamlined genomes that enable efficient growth under oligotrophic conditions. In contrast, particle-associated prokaryotes (e.g., Bacteroidetes) generally show more metabolic diversity to utilize different kinds of particulate organic matter^22^. Due to these differences, free-living and particle-associated prokaryotes should be considered as independent components, controlled by different drivers. However, only a few studies have differentiated them, and the comparisons have been mostly restricted to studying how different size fractions relate to environmental conditions^17–21,23–25^. The extent that biotic interactions with phytoplankton, protists, and viruses, influence free-living and particle-associated prokaryotes has been examined far less.

The lack of comprehensive investigations may be due, in part, to the difficulties of accessing the diversity across three domains and viruses. The emergence of high-throughput sequencing techniques allows us to identify the community composition using appropriate marker genes (e.g., SSU rRNA genes). And recent studies have shown that universal primer sets can quantitatively survey the diversity of Bacteria, Archaea, and Eukaryota in a single PCR reaction^26–28^ with high coverage of all three domains^29^. Unlike prokaryotes and eukaryotes, viruses do not contain universal genes, and thus studies of the marine viral community dynamics have tended to focus on subsets of the community, like T4-like phages using the major capsid protein-encoding gene, such as g23^16,30–36^. But increasingly, shotgun metagenomic sequencing and virus identification tools (e.g., VirSorter and VirFinder) are providing opportunities to directly reveal the distributions and dynamics of viral sequences in marine and other environments^37–40^.

**Fig. 1:**
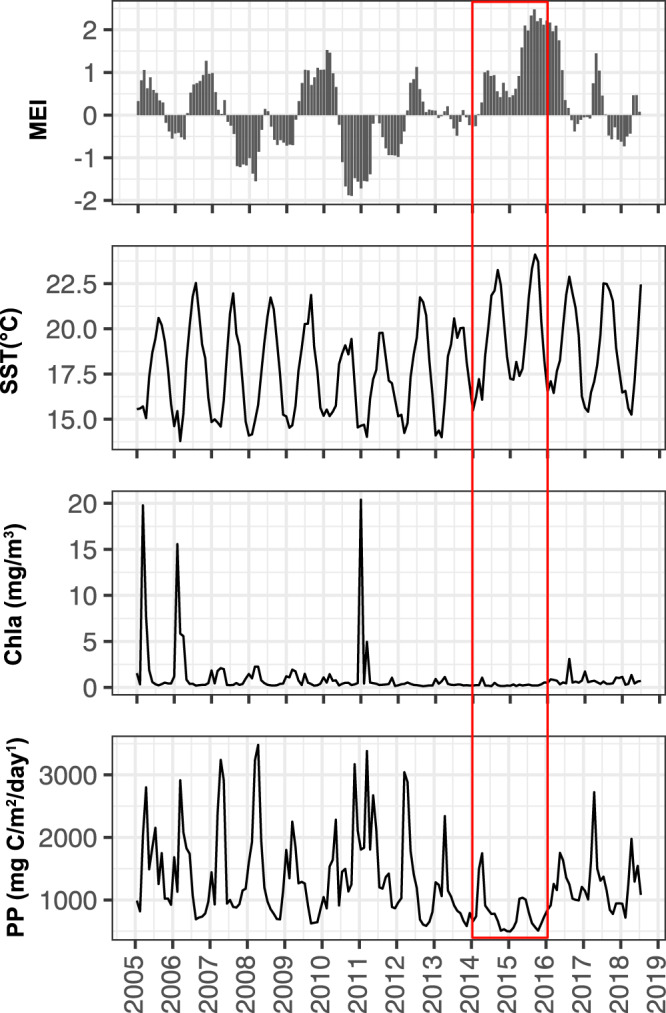
Temporal variation of multivariate ENSO index (MEI), sea surface temperature (SST), monthly average satellite chlorophyll-a concentration (Chla), and monthly average satellite primary productivity (PP) at the SPOT location. MEI index is used to characterize the intensity of the El Niño/Southern Oscillation (ENSO) event; large positive MEI values indicate El Niño conditions, whereas large negative MEI values indicate La Niña conditions. In 2014–2015 (red box), probably due to El Niño (MEI > 0) and a marine heatwave known as the “Blob”, this location experienced reduced productivity and unusually warm temperatures (particularly winters), which also persisted to a lesser extent through the end of the study. Source data are provided as a Source data file.

This study aimed to examine the effects of abiotic (environmental conditions) and biotic factors (protists, phytoplankton, and viruses) on free-living and particle-associated prokaryotic community structure from the San Pedro Ocean Time-series (SPOT) location. SPOT is located ~20 km off the coast of Southern California with ~900 m water depth and represents a subtropical mesotrophic marine ecosystem with environmental fluctuations resulting in seasonal oligotrophic conditions. Ecotypes of major taxa such as SAR11, *Synechococcus*, and *Prochlorococcus* that are seasonally abundant at SPOT are also abundant under similar temperature and nutrient conditions throughout the world^41–44^. As such, SPOT reasonably represents moderately oligotrophic to mesotrophic ocean conditions. In addition, satellite data show that this location experienced a reduced amplitude of spring phytoplankton blooms and extremely warm winters in 2014–2015 (Fig. 1), likely due to a combination of El Niño and a marine heatwave (also known as the “Blob”) in the northeast Pacific Ocean^45,46^. This marine heatwave is the strongest one ever recorded in the North Pacific, and studies have found it changed food web structures^47–50^. We thus took this opportunity to examine how warming affects seasonal and interannual variations of microbial dynamics and whether the prokaryotic community shifted in response to this warming event specifically at the community and populational levels.

In this work, we examine the temporal dynamics of free-living (0.2–1 μm) and particle-associated or larger (1–80 μm) prokaryotes, phytoplankton, protists, and viruses from 5 m depth at the SPOT location using universal SSU rRNA sequencing and viral shotgun metagenomics. With the combination of canonical analysis and variation partitioning, our results show the importance of phytoplankton and viral community in explaining the prokaryotic community composition. In addition, a populational shift within Cyanobacteria was found during the 2014/2015 warming period.

## Results

### The temporal variation of community composition

**Fig. 2:**
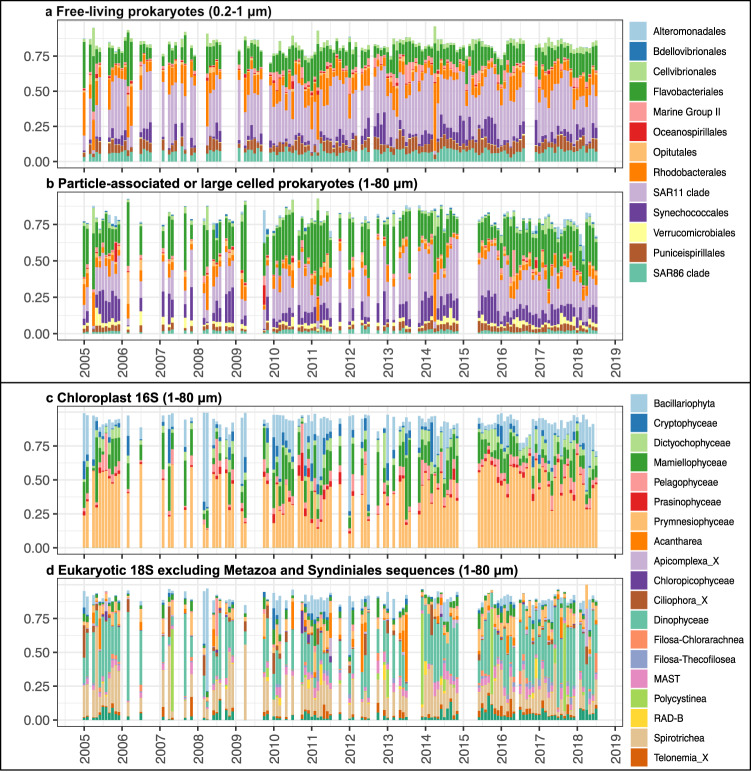
Temporal variation of the entire community composition at the SPOT location. Order level taxonomic composition of (**a**) free-living prokaryotes (0.2–1 μm), (**b**) particle-associated or large-celled (1–80 μm) prokaryotes, (**c**) chloroplast 16S (representing phototrophic eukaryotes), and (**d**) eukaryotic 18S communities (excluding Metazoa and Syndiniales sequences to better show the phytoplankton and protistan phagotrophs; see text). Note that for clarity, this shows only the most abundant taxonomic groups, with relative abundance >10% in any month. Source data are provided as a Source data file.

We explored microbial community composition between 2005 and 2018 by SSU rRNA sequencing with a universal primer set that amplifies prokaryotic 16S, chloroplast 16S (representing phototrophic eukaryotes), and 18S (representing protists) rRNA genes simultaneously, and denoising these sequences into amplicon sequence variants (ASVs) using DADA2^51^. Results showed that the most abundant prokaryotic taxa in both size fractions were SAR11, Flavobacteriales, Rhodobacterales, and Synechococcales (Fig. 2a, b). In the free-living size fraction (0.2–1 μm), SAR11 dominated most of the time, accounting for on average 31.6% of the total community composition (Fig. 2a). In the large size fraction (1–80 μm), Flavobacteriales dominated during spring blooms, whereas SAR11 dominated the rest of time (Fig. 2b). In addition, Synechococcales (cyanobacteria *Synechococcus* and *Prochlorococcus*) became particularly abundant during 2014–2015 in both size fractions.

Phototrophic eukaryotes were identified using the chloroplast 16S rRNA gene in the large size fraction (1–80 μm). Note that photosynthetic Dinophyceae (dinoflagellates) generally do not appear in the chloroplast 16S data because of unusual genetic characteristics of their chloroplasts^52^. The average relative abundance (Fig. 2c) showed that phytoplankton identified via chloroplast 16S reads were dominated by Prymnesiophyceae (coccolithophores and their relatives; 40.8%), Bacillariophyta (diatoms; 14.8%), Mamiellophyceae (mostly *Micromonas, Ostreococcus*, and *Bathycoccus*; 14%), Dictyochophyceae (6.2%), and Pelagophyceae (6.1%). Prymnesiophyceae were consistently abundant throughout the time series, whereas Bacillariophyta only peaked during spring blooms. Eukaryotic 18S reads in the large size fraction (Fig. 2d), on the other hand, were dominated by Dinophyceae (including photosynthetic and heterotrophic dinoflagellates; 28.5%), and Spirotrichea (ciliates) (18.5%). They showed distinct seasonal patterns; the proportion of Dinophyceae reads increased in summer, whereas Spirotrichea peaked during spring blooms.

### The environmental effects on free-living and particle-associated or larger prokaryotic communities

**Fig. 3:**
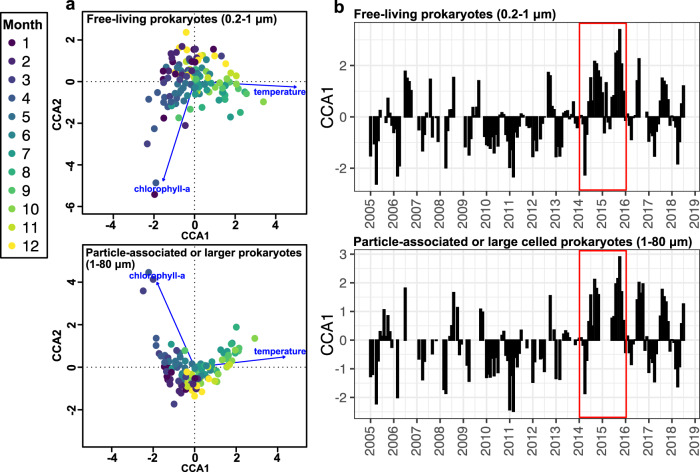
Canonical correspondence analysis (CCA) ordination illustrating the seasonal succession of the free-living and particle-associated prokaryotes, respectively. **a** CCA biplot for free-living (0.2–1 μm) and particle-associated or large-celled (1–80 μm) prokaryotes. The environmental variables analyzed are indicated as vectors, specifically water temperature and monthly average satellite chlorophyll-a concentration (chlorophyll-a). The prokaryotic communities for each sampling month are represented as circles and color-coded with the sampling months. **b** Temporal variation of the first CCA scores, which are measures of the incidence of particular community components, with higher (positive) numbers reflecting summer-like (warm, lower chlorophyll) communities, and lower (negative) numbers indicating nominal winter and spring-like communities (lower temperatures, higher chlorophyll). Note that prokaryotic communities in both size fractions were mainly summer-like in 2014–2015 (red box), corresponding to warmer years with reduced productivity and no pronounced spring bloom (Fig. 1). Source data are provided as a Source data file.

The CCA analysis showed that temperature and chlorophyll-a concentration significantly explained 4.4 and 3.8% of the variance in free-living and particle-associated or larger prokaryotic communities (Fig. 3). The first CCA axis (CCA1) represented primarily a seasonal change of community structure, which was positively correlated with temperature and negatively correlated with chlorophyll-a concentration, indicating that communities with positive CCA1 scores were summer-like (more oligotrophic), and communities with negative CCA1 scores were bloom-associated (Fig. 3). The temporal dynamics of CCA1 scores showed the seasonality of both free-living and particle-associated or larger prokaryotic communities throughout the time series, shifting from positive to negative CCA1 scores, except for 2014 and 2015, which were mostly dominated by summer-like communities the entire year.

### The roles of biotic factors in explaining free-living and particle-associated or larger prokaryotic communities

**Fig. 4:**
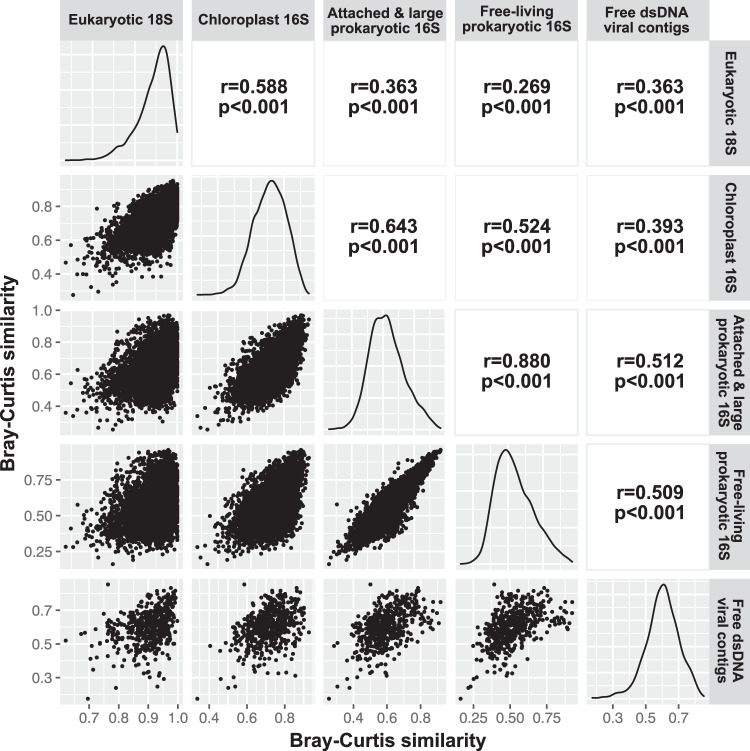
Mantel test showing correlations among different microbial components. Scatterplots of each pair of beta-diversity (i.e., Bray–Curtis similarity) are shown on the left part of the figure. The results of each Mantel test are shown on the right. The distribution of beta-diversity of each component is shown on the diagonal. The results show that free-living and particle-associated prokaryotic communities were correlated most closely (*r* = 0.880), followed by chloroplasts and attached & large prokaryotes (*r* = 0.643). Viruses were much better correlated to prokaryotes than eukaryotes (rightmost column). The statistical significance of each component was evaluated by a permutation test with 9999 permutations, and the *P*-value of all tests is 0.0001. Source data are provided as a Source data file.

**Fig. 5:**
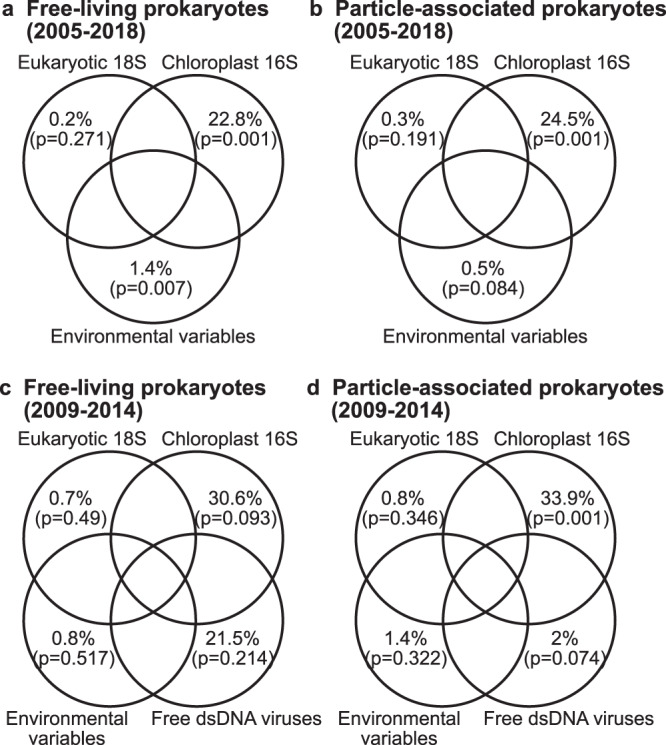
Variation partitioning of components influencing prokaryotic community composition. Percentages indicate the portion of the variance in free-living (**a**, **c**) and larger or particle-associated (**b**, **d**) prokaryotic community composition statistically explained by the respective variable. Environmental variables include temperature and chlorophyll-a. Virus data (in **c**, **d**) are available for only 2009–2014.

To test if changes in free-living and particle-associated or larger prokaryotic communities can be explained by biotic factors, including eukaryotic 18S (representing protists), chloroplast 16S (representing phototrophic eukaryotes excluding dinoflagellates), and free dsDNA viral communities, a Mantel test was used to ask the question of how closely the temporal variation in each subset of the microbial community correlated to variation in another subset (Fig. 4). The results showed that all pairs of beta-diversity matrices were significantly correlated (*p* < 0.001), though the *r* values varied greatly (0.27–0.88). The strongest correlations were between free-living and particle-associated prokaryotic communities (*r* = 0.88), followed by chloroplasts and particle-associated prokaryotes (*r* = 0.64), eukaryotes and chloroplasts (*r* = 0.59), chloroplasts and free-living prokaryotes (*r* = 0.52), and viruses and both free and particle-associated prokaryotes (*r* = 0.51). However, these correlations may be caused in part by seasonal reoccurring patterns, such as seasonal temperature fluctuations and spring phytoplankton blooms. Thus, partial CCA (pCCA) analysis was used to remove these effects (i.e., temperature and chlorophyll-a, which both have broad effects on each subset of the microbial community) (Table 1). The pCCA analysis indicated that all the components of the microbial community (i.e., eukaryotic 18S, chloroplast 16S, and free-dsDNA viral contigs) significantly explained the temporal variation of free-living and particle-associated prokaryotic community structure after removing the effects of temperature and chlorophyll-a (*p* < 0.05). Notably, chloroplast 16S and free dsDNA viruses were strong predictors of free-living prokaryotic communities, explaining 27.4% and 28.1% of the variation. For particle-associated or larger prokaryotes, chloroplast 16S communities significantly predicted the community composition, explaining 30.9% of the variation, against 9.3% attributed by free dsDNA viruses. The eukaryotic 18S communities (which have phytoplankton as well as heterotrophic protists), on the other hand, only explained 2.7% and 5.7% of the variation. After testing the significance of each component, we used variation partitioning to determine the relative importance of each component in explaining the temporal variation of free-living and particle-associated prokaryotic community structure (Fig. 5). Note that a subset of the time series between 2009 and 2014 was used to include the free dsDNA viruses into the analysis (when such data were available). The results showed that for the whole time series (2005–2018), free-living and particle-associated prokaryotic communities were significantly explained by the unique effects of chloroplast 16S and environmental variables. The unique effect of chloroplast 16S explained 22.8% and 24.5% of the variation, whereas the unique environmental effect was only marginally significant and explained <1.5% of the variation. The unique effects of eukaryotic 18S were insignificant for both size fractions. For the subset of the time series between 2009 and 2014, the unique effects of chloroplast 16S were significant for particle-associated or larger prokaryotes, but not for free-living ones.

**Table 1:** Portion of the variance of free-living and particle-associated or larger prokaryotic 16S community explained by each component after partitioning out the environmental effect (i.e., temperature and chlorophyll-a) using partial CCA

	Free-living prokaryotes		Particle-associated prokaryotes	
	% Variance	*P*-value	% Variance	*P*-value
Phytoplankton (chloroplast 16S) community	27.4%	0.001	30.9%	0.001
Protists (18S) community	2.7%	0.027	5.7%	0.015
Free dsDNA viral contig community	28.1%	0.001	9.3%	0.001

The statistical significance of each component was evaluated by a permutation test with 999 permutations.

### Warming effects on prokaryotic community composition at the community and populational levels

To examine how community composition shifted in response to the 2014/2015 warming event, we focused on two different levels: the community level and the populational level. At the community level, only Synechococcales (Cyanobacteria) in the particle-associated size fraction significantly increased in 2014–2015 (Fig. 2b, Kruskal–Wallis test, *p* < 0.001), yet other major taxonomic groups (SAR11, Flavobacteriales, and Rhodobacterales) did not change significantly in their relative abundances (Fig. 2a, b, Kruskal–Wallis test, *p* > 0.001), indicating that only a portion of the community was significantly affected by the warming event.

**Fig. 6:**
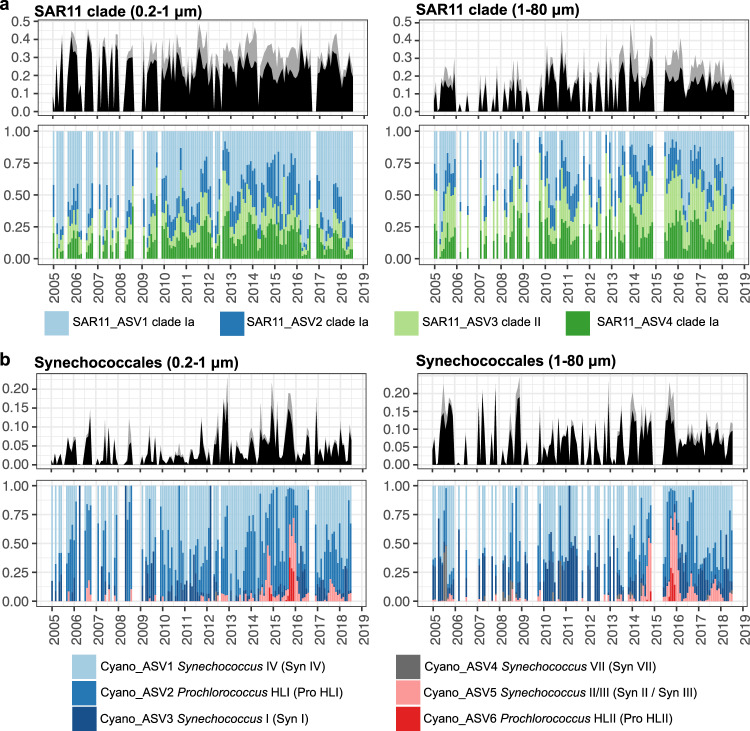
Relative abundance of major ASVs within SAR11 and Synechococcales cyanobacteria. Gray areas in the top graphs represent rarer ASVs of the clades not specifically included in the lower graphs. Note the appearance of *Prochlorococcus* HLII clade (red) only in the warmest years, 2014–2015. Source data are provided as a Source data file.

At the populational level, we examined whether ASV composition within the major taxonomic groups shifted in 2014–2015. For SAR11, 4 major ASVs were persistently abundant (Fig. S1) throughout the overall time series, accounting for >50% of total SAR11 sequences in both size fractions (Fig. 6a). SAR11_ASV1 (clade Ia) showed maximum abundances during spring, whereas SAR11_ASV2 (clade Ia), SAR11_ASV3 (clade II), and SAR11_ASV4 (clade Ia) peaked in summer. Among these ASVs, SAR11_ASV1 (clade Ia) in the free-living size fraction significantly decreased in the 2014/2015 warming period (Kruskal–Wallis test, *p* < 0.001), whereas SAR11_ASV2 (clade Ia) significantly increased in 2014–2015 (Kruskal–Wallis test, *p* < 0.001).

For Synechococcales (Cyanobacteria), 6 major ASVs either persistently occurred or had become >5% at least once (Fig. S1) over the entire time series, and they contributed >42% of total Synechococcales (Cyanobacteria) sequences (Fig. 6b). Among these, Cyano_ASV1 (Syn IV), Cyano_ASV2 (Pro HLI), and Cyano_ASV3 (Syn I) were the dominant ASVs most of the time. During the 2014/2015 warming period, Cyano_ASV5 (Syn II/III) and Cyano_ASV6 (Pro HLII) significantly increased in both size fractions (Kruskal–Wallis test, *p* < 0.001), and Cyano_ASV2 (Pro HL) significantly increased in particle-associated size fraction (Kruskal–Wallis test, *p* < 0.001).

## Discussion

This study used SSU rRNA amplicon and shotgun metagenomic sequencing to survey the large majority of microbial food web members (i.e., free dsDNA viruses, free-living and particle-associated prokaryotes, phytoplankton, and protists). With a combination of canonical analyses and variation partitioning, our results determined that the seasonal dynamics of free-living and particle-associated prokaryotes were significantly explained, statistically, by biotic interactions with phytoplankton (i.e., chloroplast 16S) and free dsDNA viral communities (i.e., viral contig abundance). Although correlation does not imply causation, the biological interactions we know of from prior marine research lead us to believe that prokaryotic community composition may be driven to a large extent by substrates provided by phytoplankton, suggesting a significant element of bottom-up control^53–58^. However, the cause-and-effect relationship between viruses and prokaryotes is potentially more fluid, and we consider two non-mutually-exclusive explanations, that may both apply, considering the large number of virus-host pairs and varied, dynamic conditions over the several years studied. One is that many viruses may follow their hosts closely, because these obligate parasites require their hosts to reproduce, and under some circumstances the viruses and their hosts can coexist indefinitely^59–61^. By this thinking, changes in the viral community would be largely responding to changes in prokaryotes caused by other factors. The second potential explanation is that for some of the prokaryotes, their composition could in part be driven by viruses through viral lysis and non-steady-state “kill the winner” dynamics, where a high density of a blooming host prokaryote leads to the rapid spread of a viral infection that knocks down the host population (i.e., top-down control). Our current analyses cannot directly point to the dominance of either mechanism. However, the previous study has shown that SPOT viral dynamics are dominated by largely steady populations at the 98% nucleotide level, plus rapid changes of viral strains within populations^39^. This is consistent with the hypothesis that viruses are primarily following their hosts at the species level, but viruses are often driving strain-level changes in host composition and vice versa.

Viral infection is generally believed to be strongly taxon-specific, and it plays an important role in the microbial loop. However, previous studies have only focused on the free-living size fraction. How viruses affect particle-associated marine prokaryotes has rarely been discussed. Given the differences in lifestyle, we expect that infection patterns are different between free-living and particle-associated prokaryotes. For free-living prokaryotes, viruses lysing one cell may have a limited ability to infect another cell, as free-living prokaryotes are widely dispersed in the environment, and susceptible hosts to each viral type (only a small fraction of the community) can be hard to encounter. Particle-associated prokaryotes, on the other hand, are often largely clonal (spreading rapidly from initially infecting pioneer cells) and geographically restricted to the particle, which give lysing viruses ample opportunities to infect adjacent cells. Our results show that the free dsDNA viral community significantly explained 28.1% and 9.3% of the variation for free-living and particle-associated prokaryotes, respectively, suggesting that free dsDNA viruses may follow the free-living prokaryotes more closely. We can currently only speculate why the correlations between free dsDNA viruses and particle-associated prokaryotes were relatively weaker, such as the possibility that free viruses are dominated by those infecting the free-living planktonic bacteria, and less well represent those infecting prokaryotes on particles.

It is interesting that, although phytoplankton (via chloroplast 16S) had a strong influence on prokaryotes, we found insignificant effects of overall 18S communities on prokaryotic community structure, which suggests that grazing by particular protists may not selectively and predictably affect prokaryotic communities in both size fractions (Fig. 5 and Table 1). The 18S communities at SPOT were dominated by Dinophyceae (i.e., dinoflagellates) and Spirotrichea (i.e., ciliates) (Fig. 2d), though we must keep in mind that it is likely that some of that apparent dominance is due to high 18S rRNA copy number^52,62,63^, and not necessarily dominant biomass. These groups, and several others reported in our results and known to be phagotrophs or mixotrophs (e.g., Mamiellophyceae and Prymnesiophyceae), with members that are known to effectively graze on various microorganisms and remove their biomass out of the microbial loop. Previous studies based on culture experiments have shown that various flagellates and ciliates generally prefer larger prey and/or prey with high activity, and only some effectively graze free bacterioplankton^10,64–66^. In addition, the ability of prokaryotes to develop grazing resistance mechanisms (e.g., motility and toxin release, or survival in vacuoles) can protect some prokaryotes from some grazing losses^67^, and this is hard to predict from ASV data. As different prokaryotic taxonomic groups have distinct properties, protists could selectively graze on particular prokaryotic populations. However, our results suggest that protist grazing is not sufficiently taxa-selective to show up as controlling prokaryote composition. This is in accordance with previously studies^68–70^.

As our long-term sampling captured one of the strongest known marine heatwaves in the North Pacific between 2014 and 2015 (Fig. 1), we were able to examine how it affected prokaryotic community composition at different levels. Our results show that warming did not influence the overall taxonomic composition, except for Synechococcales cyanobacteria, which significantly increased in the larger and particle-associated size fraction (Fig. 2). However, when we examined the warming effects down to the populational level, compositional shifts (ecotype shifts) emerged (Fig. 6). Together, our findings suggest that warming did not influence prokaryotic community dynamics at a broader level but changed populational structures at a finer level. Specifically, warming resulted in populational shifts from cold-water ecotypes to warm-water ecotypes.

For SAR11, 4 major ASVs displayed seasonal variation (Fig. 6). SAR11_ASV1 (SAR11 clade Ia) dominated during spring, whereas SAR11_ASV2 (SAR11 clade Ia), SAR11_ASV3 (SAR11 clade II), and SAR11_ASV4 (SAR11 clade Ia) peaked in summer. Previous studies have subdivided SAR11 clade Ia into cold-water (Ia.1) and warm-water (Ia.3) ecotypes, which have distinct latitudinal distributions^41^ and seasonal patterns^71^. Although the SAR11 ASVs in this study were not identified to the finest level, their seasonal patterns imply that SAR11_ASV1 may be cold-water ecotypes (Ia.1), and SAR11_ASV2 and SAR11_ASV4 might be warm-water ecotypes (Ia.3). Given this assumption, warming resulted in a decrease in the relative abundance of cold-water ecotypes (SAR11_ASV1) and an increase of warm-water ecotype (SAR11_ASV2). In addition, we found that SAR11_ASV3 (SAR11 clade II) was enriched in the large size fraction, especially in 2014–2015, which is surprising. SAR11 members are widely believed to be free-living bacteria, and to our knowledge, such significant enrichment on particles has not been previously reported. Our finding suggests that some members of SAR11 clade II might have a previously unknown niche on particles.

Synechococcales cyanobacteria were found to exhibit compositional changes at the ASV level during warming. There were 6 major ASVs dominating the cyanobacterial population, and they were identified as different ecotypes (Fig. 6). Synechococcales can be further classified into genera *Prochlorococcus* and *Synechococcus*, which are different in size, photosynthetic pigments, and general ecological preferences. *Prochlorococcus* tends to occur in more oligotrophic conditions, is smaller (0.6–0.8 μm), and possesses divinyl chlorophyll a and b, whereas *Synechococcus* tends to live in more mesotrophic conditions, is larger (0.6–2 μm) and has phycobilisomes^72,73^. Within each genus, several ecotypes are reported to have distinct niches in light, temperature, and iron requirements^42–44,74,75^. Our results showed that the warming event in 2014–2015 resulted in a shift from cold-water ecotypes (i.e., Cyano_ASV1 (Syn IV), Cyano_ASV2 (Pro HLI), and Cyano_ASV3 (Syn I)) to warm-water and more oligotrophic ecotypes (i.e., Cyano_ASV5 (Syn II/III) and Cyano_ASV6 (Pro HLII)). Similar patterns were also observed in the MICRO time series at Newport Pier, California, which is adjacent to a beach and 44 km away from the SPOT location^76^. This suggests that shifts in the cyanobacterial population due to warming occurred at a regional scale from the coast to more offshore locations of Southern California Bight.

In contrast to SAR11 and Cyanobacteria, Flavobacteriales and Rhodobacterales only showed modest changes in their ASV composition in 2014–2015 (Fig. S2). These two taxonomic groups have been described as major components of bloom-associated communities. Flavobacteriales are specialized to degrade complex organic matter, whereas Rhodobacterales consume primarily low molecular weight phytoplankton metabolites. It was expected that these two bloom-associated groups might diminish dramatically in 2014–2015 since the environmental condition was relatively oligotrophic. However, Flavobacteriales and Rhodobacterales remained the same in the warming period (Fig. 2). Previous studies have reported that experimental warming increased the relative abundance of bloom-associated communities by increasing their chemotaxis ability and metabolism^77,78^, suggesting that bloom-associated prokaryotes may be favored by warming since their ability to detect, pursue, and utilize phytoplankton-derived substrates are enhanced by warmer temperature.

Overall, we found that the warming of 2014–2015 resulted in an increase in the relative abundance of warm-water ecotypes. There are two potential causes of this shift in community composition. First, pre-existing warm-water ecotypes can gain a competitive advantage under warming conditions and thus outcompete cold-water ecotypes. Second, the warm-water ecotypes may undergo geographic range expansion from more oligotrophic offshore warm water into the Southern California Bight^79^. Our results are consistent with both of these non-exclusive scenarios. For example, the major ASVs of SAR11 persistently occurred at the SPOT location throughout the whole time series (Fig. 6a), indicating that they belong to the local community. Thus, the shift in SAR11 ecotypes to year-round warmer-water ecotypes in an El Niño year is a potentially expected result from competition. However, Cyano_ASV6 (Pro HLII) was not observed at SPOT in the several years before 2014, and it appeared and suddenly peaked in 2014–2015 (Fig. 6b); this suggests Cyano_ASV6 (Pro HLII) was probably introduced and took hold due to range expansion from more oligotrophic offshore Pacific waters.

In summary, our study revealed the bottom-up control of phytoplankton communities on free-living and particle-associated marine prokaryotes and how closely free dsDNA viruses were infecting and following their hosts, especially the free-living ones. Protistan communities as a whole, however, did not affect prokaryotic communities, at least in a statistical sense. Overall, our results suggested the importance of bottom-up control on prokaryotic community structure, yet whether the community changes influence their functioning remains unknown and needs further investigation. This correlation-based analysis now shows the overall statistical relationships between each major subset of the microbial community, and future analyses could examine much more specific interactions between organisms using other statistical and network analyses, such as empirical dynamic modeling (EDM)^80^. In addition, we found that warming resulted in significant changes in prokaryotic populations, especially for cyanobacteria, shifting from cold-water ecotypes to warm-water ecotypes. Although this was not a permanent regime shift due to general warming, it is the kind of observation that supports the value of continuously monitoring the impacts of climate change through long-term time series efforts.

## Methods

### Sample collection and DNA extraction

Samples were collected monthly from 5 m at San Pedro Ocean Time-series (SPOT) location during 2005–2018. Approximately 12 L of seawater was sequentially filtered through an 80-μm mesh, a 1-μm A/E filter (Pall, Port Washington, NY; nominal pore size 1.2 μm), and a 0.2-μm Durapore filter (ED Millipore, Billerica, MA). Filters were stored at −80 °C until DNA extraction. Durapore filters (0.2–1 μm) were used for free-living prokaryotic community analysis, and A/E filters (1–80 μm size fraction) were used to analyze phytoplankton, protists, and particle-associated or large-celled prokaryotic communities (as the 1 μm AE filters capture not only those attached to larger particles but also some free-living prokaryotic cells). DNA was extracted from the Durapore filters using a hot SDS, phenol/chloroform/isoamyl alcohol, ethanol precipitation extraction protocol described at 10.17504/protocols.io.dmi44d. DNA was extracted from the A/E filters using a NaCl/CTAB bead-beating extraction protocol as described by Kim et al^81^ with slight modification by adding an ethanol precipitation step after lysis to reduce the volume of crude extract, which helps minimize DNA loss during the subsequent purification. The detailed protocol was described at 10.17504/protocols.io.ewov1oqeklr2/v1.

### Environmental data

Monthly average sea surface temperature, surface chlorophyll-a concentrations, and primary productivity were downloaded as satellite data from the Coastwatch browser website (https://coastwatch.pfeg.noaa.gov/erddap/index.html). The multivariate ENSO index (MEI) was downloaded from the National Oceanographic and Atmospheric Administration (NOAA).

### 16S/18S PCR and sequencing

To pool multiple samples in a single Illumina paired-end sequencing platform, a dual-index sequencing strategy was used with the forward primer *A*-*I*-NNNN-barcode-515Y (*A*-*I*-NNNN-barcode-GTGYCAGCMGCCGCGGTAA) and reverse primer *A*-index-*I*-926R (*A*-index-*I*-CCGYCAATTYMTTTRAGTTT), where *A* is the Illumina sequencing adapter, *I* is the Illumina primer, and barcode and index are sample-specific tag (5-bp barcode and 6-bp index). 515Y/925 R primer pairs target the V4-V5 hyper-variable region of the 16S/18S rRNA genes. All DNA samples were amplified using the same conditions described at 10.17504/protocols.io.vb7e2rn. PCR products were cleaned using 0.8X Ampure XP magnetic beads (Beckman Coulter). Purified PCR products from samples were pooled in equal amounts and then sequenced on Illumina HiSeq 2500 in PE250 mode and MiSeq PE300. For each sequencing run, multiple blanks (i.e., PCR water) and two versions of mock communities (even and staggered) were included as internal controls, meaning they were amplified, cleaned, and sequenced as environmental samples with the same conditions. This way, results from different sequencing runs were comparable without significant bias and contamination^26,27,82^.

### Sequence analysis

Sequences were demultiplexed by forward barcodes and reverse indices allowing no mismatches using QIIME 1.9.1 split_libraries_fastq.py^83^. The fully demultiplexed forward and reverse sequences were then split into per-sample fastq files using QIIME 1.9.1 split_sequence_file_on_sample_ids.py.

Demultiplexed amplicon sequences were trimmed with cutadapt (v2.3) implemented in QIIME2 (v2019.4), discarding any sequence pairs not containing the forward or reverse primer (error rate set to 0.2). Amplicon sequences were then split into 16S and 18S pools using bbsplit.sh from the bbtools v38.22 (http://sourceforge.net/projects/bbmap/) against curated 16S/18S databases derived from SILVA 132^84^ and PR2 4.10.0^85^. The 16S and 18S amplicons were then analyzed in parallel to amplicon sequence variants (ASVs) using DADA2 (v1.10)^51^ implemented in QIIME2 (v2019.4)^86^. 16S ASVs were classified with qiime2 classify-sklearn plugin against the SILVA 132 database^84^. 16S ASVs identified as Mitochondria were removed. Then, the ASV table was subdivided into prokaryotic 16S ASV table and chloroplast 16S ASV table (including all 16S ASV identified as Chloroplast). Chloroplast 16S ASVs were further classified against PhytoRef database^87^. 18S ASVs were assigned against the PR2 4.10.0 database^85^. Metazoa and Syndiniales ASVs were removed from the final 18S ASV table because they represent organismal fragments, eggs, juveniles, and parasites that are either too stochastically variable or thought to not directly interact with prokaryotes.

### Free dsDNA viral community

The same seawater samples collected monthly from the 5 m depth at SPOT during 2009–2014 were used for viral community analysis using metagenomic sequences from the 0.02–0.22 μm size fraction^39^. 19,907 putative viral contigs were generated from the metagenome assembly, and read recruitment to those contigs was used to assess their relative abundance, as previously reported^39^

### Statistical analyses

The prokaryotic 16S ASV table was subdivided into two groups, free-living (0.2–1 μm) and particle-associated (1–80 μm) prokaryotes according to size fraction. The chloroplast 16S ASV table from the 1–80 μm size fraction was used to represent the phytoplankton (noting it misses most dinoflagellates; Needham and Fuhrman^52^). The 18S ASV table from the 1–80 μm size fraction was used to represent all protists. The dominant ASVs (i.e., >0.5% of relative abundance occurred at least once throughout the time series) were selected for further analysis to reduce the noise associated with stochastic variations of the rarer species.

This study surveyed all the major components of marine microbial food webs, including prokaryotes, phytoplankton, protists, and viruses. All these organism types are expected to interact with each other, yet the mechanisms behind some of these interactions remain unclear. The relatively well-studied interactions are the effects of phytoplankton, protists, and viruses on prokaryotes via bottom-up and top-down controls. Even though we recognize there may also be less understood effects of prokaryotes on the phytoplankton^88^ and other protists, and of prokaryote hosts on their viruses (i.e. viruses following their hosts), we focus on the well-known interactions and used canonical analyses with prokaryotic communities always in the dependent role whereas the environmental data, phytoplankton (chloroplast 16S), protists (eukaryotic 18S), and viruses (dsDNA virus) communities are in explanatory roles. The compositional data were Hellinger transformed^89^, and environmental variables were standardized prior to canonical analyses. Detrended correspondence analysis (DCA) was first used to determine the appropriate response model (canonical correspondence analysis (CCA) or redundancy analysis (RDA)) for prokaryotic communities^90^. The longest gradient lengths from DCA determine a suitable canonical analysis for a given community composition (CCA for >3; RDA for <3). The results showed that CCA worked better for prokaryotic communities, thus CCA was directly used to relate standardized environmental variables to prokaryotic communities^91^. For the compositional data (chloroplast 16S, 18S, and dsDNA virus), a two-step (indirect) approach was used because CCA breaks down when the number of species (i.e., ASVs/viral contigs) is larger than the number of sampling months in our case. Thus, we applied correspondence analysis (CA) to the compositional data (chloroplast 16S, 18S, and dsDNA virus) and took the first few CA axes which retain at least 70% of variation as explanatory variables. Then, only significant CA axes selected by a stepwise forward selection procedure were used for the final CCA model. In addition, partial CCA (pCCA) was used to remove the effects of environmental variables. To further determine how much of the variation in prokaryotic composition was solely explained by each component, variation partitioning analysis was used here^92^. All statistical analysis and visualization were conducted with R (v4.1.0) using ade4 (v1.7.18), vegan (v2.5.7), gpplot2 (v3.3.6), and GGally (v2.1.2) packages.

### Reporting summary

Further information on research design is available in the Nature Portfolio Reporting Summary linked to this article.

## Supplementary information

Supplementary Information

Reporting Summary

## Data Availability

Raw sequence data have been deposited to the EMBL database under accession codes PRJEB48162 and PRJEB35673. Source data are provided with this paper.

## Source data

Source Data

## Code availability

Scripts necessary to reproduce the sequencing analyses are available at https://github.com/jcmcnch/eASV-pipeline-for-515Y-926R and archived on Zenodo (10.5281/zenodo.7340378). The R scripts of all the statistical analyses are available via Figshare (10.6084/m9.figshare.21363153.v1).
