# Supplementary material for: Effects of phytoplankton, viral communities, and warming on free-living and particle-associated marine prokaryotic community structure
Source: Nat Commun. 2022 Dec 23;13:7905. doi: 10.1038/s41467-022-35551-4 (PMC9780322; doi:10.1038/s41467-022-35551-4)

Supplementary information

Effects of phytoplankton, viral communities, and warming on free-living and particle-associated marine prokaryotic community structure

Yi-Chun Yeh[1] and Jed A. Fuhrman[1*]

[1]Department of Biological Sciences, University of Southern California, Los Angeles, California 90089-0371, USA

Correspondence:

Jed A. Fuhrman

Department of Biological Sciences, University of Southern California, Los Angeles, California 90089-0371, USA

Email: fuhrman@usc.edu

Figure S1. Occurrence frequency and abundance of ASVs within each major taxonomic group. The mean relative abundance (left) and maximum relative abundance (right) are plotted against the occurrence frequency (the proportion of communities/times in which each ASV was detected). Rhodobacterales and SAR11 were all dominated by a few major ASVs that occurred >75% of the time. Flavobacteriales and Synechococcales were dominated by major ASVs that occurred >75% of the time and a few ASVs that were occasionally abundant (>5%).

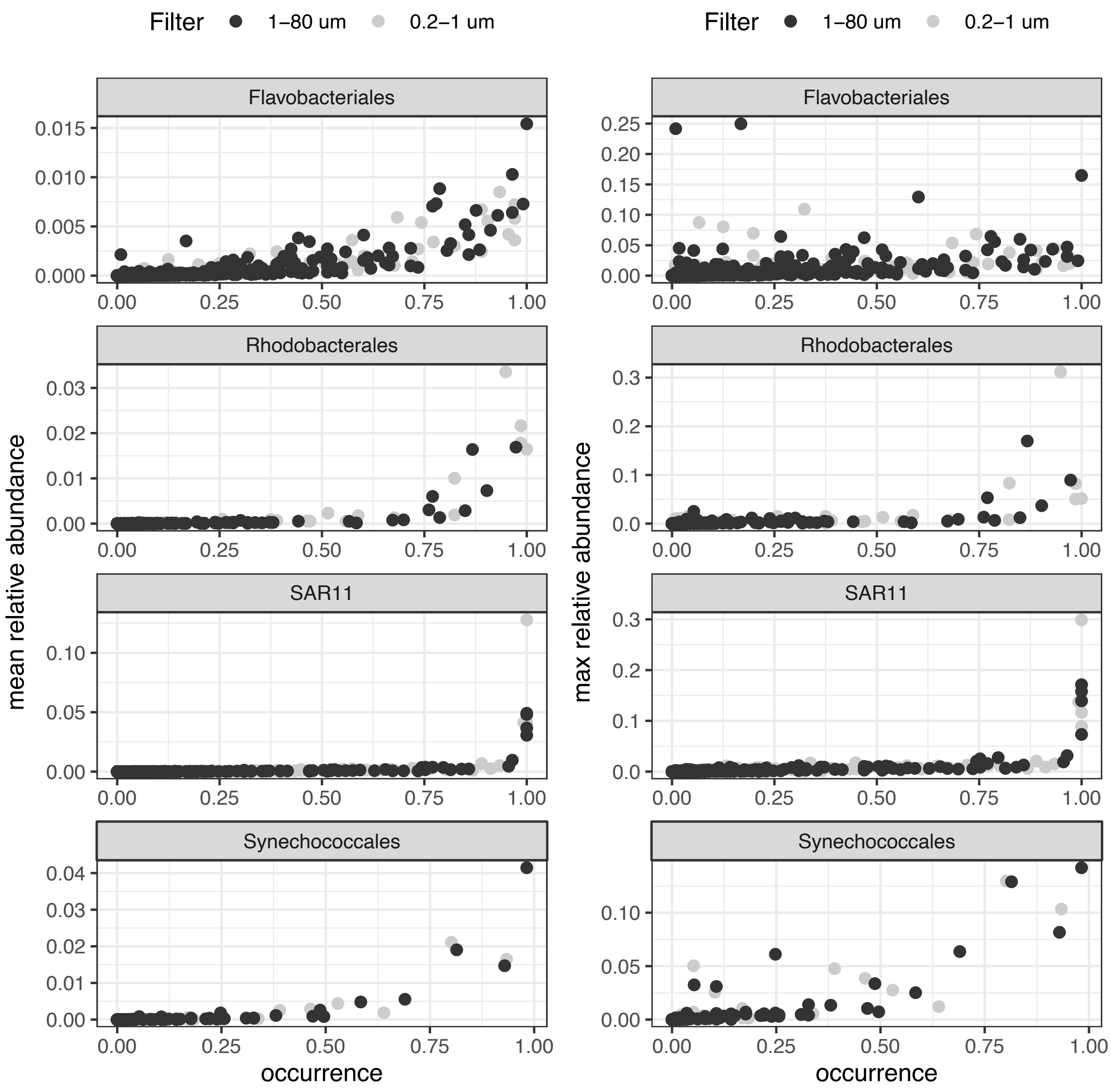

Figure S2. Relative abundance of major ASVs within Rhodobacterales and Flavobacteriales. Grey areas in the top graphs represent rarer ASVs of the clades not specifically included in the lower graphs.

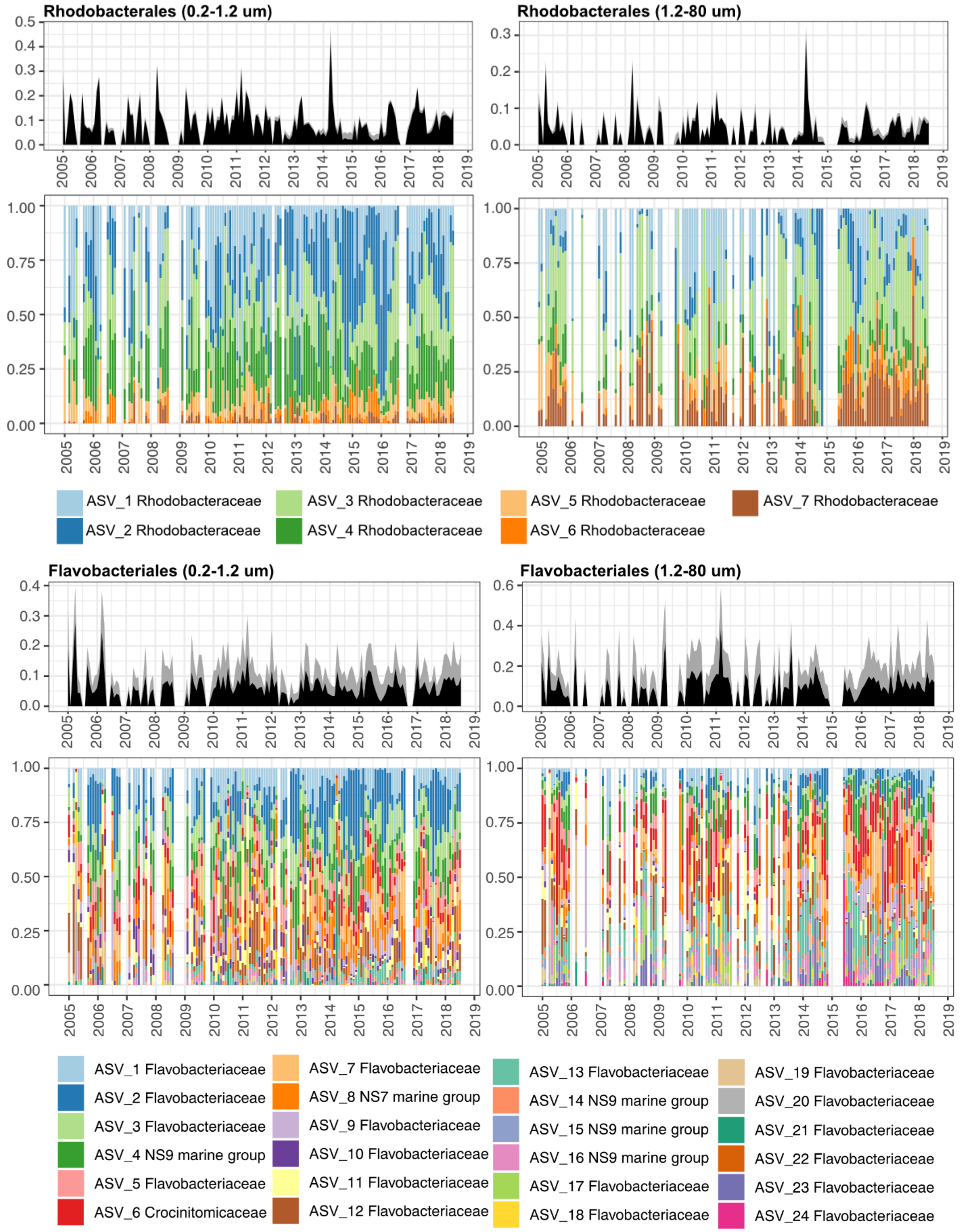

Supplement: Supplementary file 1 — Supplementary Information [file 41467_2022_35551_MOESM1_ESM.pdf]
