# Supplementary material for: Effects of phytoplankton, viral communities, and warming on free-living and particle-associated marine prokaryotic community structure
Source: Nat Commun. 2022 Dec 23;13:7905. doi: 10.1038/s41467-022-35551-4 (PMC9780322; doi:10.1038/s41467-022-35551-4)
Supplement: Supplementary file 2 — Reporting Summary [file 41467_2022_35551_MOESM2_ESM.pdf]

nature portfolio

Corresponding author(s): Jed Fuhrman

Last updated by author(s): Nov 21, 2022

Reporting Summary

Nature Portfolio wishes to improve the reproducibility of the work that we publish. This form provides structure for consistency and transparency in reporting. For further information on Nature Portfolio policies, see our Editorial Policies and the Editorial Policy Checklist.

Statistics

For all statistical analyses, confirm that the following items are present in the figure legend, table legend, main text, or Methods section.

n/a	Confirmed	
x		The exact sample size (n) for each experimental group/condition, given as a discrete number and unit of measurement
	x	A statement on whether measurements were taken from distinct samples or whether the same sample was measured repeatedly
	x	The statistical test(s) used AND whether they are one- or two-sided<br>*Only common tests should be described solely by name; describe more complex techniques in the Methods section.*
	x	A description of all covariates tested
	x	A description of any assumptions or corrections, such as tests of normality and adjustment for multiple comparisons
	x	A full description of the statistical parameters including central tendency (e.g. means) or other basic estimates (e.g. regression coefficient) AND variation (e.g. standard deviation) or associated estimates of uncertainty (e.g. confidence intervals)
	x	For null hypothesis testing, the test statistic (e.g. F, t, r) with confidence intervals, effect sizes, degrees of freedom and P value noted<br>*Give P values as exact values whenever suitable.*
x		For Bayesian analysis, information on the choice of priors and Markov chain Monte Carlo settings
	x	For hierarchical and complex designs, identification of the appropriate level for tests and full reporting of outcomes
x		Estimates of effect sizes (e.g. Cohen's d, Pearson's r), indicating how they were calculated

Our web collection on statistics for biologists contains articles on many of the points above.

Software and code

Policy information about availability of computer code

Data collection: No software was used for data collection.

Data analysis: Raw sequence data were trimmed with cutadapt (v2.3) implemented in QIIME2 (v2019.4) and split into 16S and 18S pools using bbsplit in bbtools (v38.22) against curated 16S/18S databases derived from SILVA 132 and PR2 4.10.0. The 16S and 18S rRNA sequences were then denoised into amplicon sequence variants (ASVs) using DADA2 (v1.10) implemented in QIIME2 (v2019.4). 16S ASVs were classified against the SILVA 132 database. Chloroplast 16S ASVs were further classified against PhytoRef database. 18S ASVs were assigned against the PR2 4.10.0 database. Scripts necessary to reproduce the sequencing analyses are available at https://github.com/jcmcnch/eASV-pipeline-for-515Y-926R and archived on Zenodo (https://doi. org/10.5281/zenodo.7340378).

All statistical analyses and visualization were conducted with R v.4.1.0 using ade4 v1.7.18, vegan v2.5.7, ggplot2 v3.3.6, and GGally v2.1.2 packages. The R scripts of all the statistical analyses are available via Figshare (https://doi.org/10.6084/m9.figshare.21363153.v1).

For manuscripts utilizing custom algorithms or software that are central to the research but not yet described in published literature, software must be made available to editors and reviewers. We strongly encourage code deposition in a community repository (e.g. GitHub). See the Nature Portfolio guidelines for submitting code & software for further information.

Data

Policy information about availability of data

All manuscripts must include a data availability statement. This statement should provide the following information, where applicable:

- Accession codes, unique identifiers, or web links for publicly available datasets
- A description of any restrictions on data availability
- For clinical datasets or third party data, please ensure that the statement adheres to our policy

Raw sequence data have been deposited to the EMBL database under accession codes PRJEB48162 [https://www.ncbi.nlm.nih.gov/bioproject/PRJEB48162] and PRJEB35673 [https://www.ncbi.nlm.nih.gov/bioproject/?term=PRJEB35673].

Field-specific reporting

Please select the one below that is the best fit for your research. If you are not sure, read the appropriate sections before making your selection.

☐ Life sciences ☐ Behavioural & social sciences ☒ Ecological, evolutionary & environmental sciences

For a reference copy of the document with all sections, see nature.com/documents/nr-reporting-summary-flat.pdf

Ecological, evolutionary & environmental sciences study design

All studies must disclose on these points even when the disclosure is negative.

Study description	This is a 14-year long time term study, which we observed the temporal dynamics of the entire microbial community (including protists, phytoplankton, prokaryotes, and free viruses) at the monthly scale using SSU rRNA sequencing and viral metagenomics. Seawater was collected monthly from 5m depth at the San Pedro Time-series (SPOT) location between 2005-2018 without experimental manipulation and with no replicates.
Research sample	Seawater samples were collected monthly in order to capture the seasonality of the system. Each month, about 12 L of surface seawater was sequentially filtered through an 80-um mesh, a 1-um A/E filter, and a 0.2-um Durapore filter. In addition, 1L of the same seawater was filtered through an 80-um mesh, a 0.2 um Sterivex filter, and a 0.02 um Anotop filter.<br><br>A/E filters (1-80 um) represent particle-associated prokaryotes and eukaryotes (protists and phytoplankton). Durapore filters (0.2-1 um) represent most of the free-living prokaryotes, including bacteria and archaea. Anotop filters (0.02-0.2 um) represent free marine viruses.
Sampling strategy	About 12L of seawater samples were collected monthly. Previous DNA extractions have shown that 12L of seawater samples are sufficient to get enough DNA for the molecular work. As this is a long-term time series study, we have sampled the same volume to minimize sampling bias throughout the study.
Data collection	Seawater samples were collected by a SeaBird CTD-General Oceanic Rosette assembly with Go-Flo bottles launched from the research vessel Yellowfin by Troy Gunderson and Fuhrman Lab members. Seawater filtration were performed the day of the cruise by Fuhrman Lab members.<br><br>Environmental variables (including sea surface temperature, chlorophyll-a concentrations, and primary productivity) were downloaded from the Coastwatch browser website by Yi-Chun Yeh. Multivariate ENSO index (MEI) was download from the National Oceanographic and Atmospheric Administration (NOAA) by Yi-Chun Yeh.<br><br>Prokaryotic and eukaryotic community analyses were conducted by Yi-Chun Yeh, and the procedure has been described in the main text. Free dsDNA viral community analysis was conducted by J. Cesar Ignacio-Espinoza, and the procedure has been published and cited in the main text.
Timing and spatial scale	Samples were collected at the San Pedro Ocean Time-series (SPOT) location, which is 20km off the coast of the southern California. As coastal ecosystems provide critical ecosystem services, the proximity of the SPOT location provides an opportunity to study the human impact on the marine environment.<br><br>This sampling location is a seasonal subtropical marine ecosystem, so we collected seawater samples monthly to capture the seasonality of the system. However, due to weather issues or sampling failure, there are 27 gaps/missing months (Feb-2005, Jul-2005, Aug-2005, May-2006, Jun-2006, Nov-2006, Dec-2006, Jan-2007, Mar-2007, Jul-2007, Oct-2007, Jan-2008, Feb-2008, Mar-2008, Sep-2008, Oct-2008, Nov-2008, Dec-2008, Jan-2009, Mar-2009, Oct-2009, Nov-2009, Apr-2012, Aug-2012, Sep-2016, Oct-2016, Nov-2016).
Data exclusions	Durapore samples collected at Sep-2016, Oct-2016, and Nov-2016 were excluded from the analysis due to contaminations.<br>A/E samples collected at Jan-2015, Feb-2015, Mar-2015, Apr-2015, May-2015 were excluded from the analysis due to contaminations.
Reproducibility	As this study is a long-term observation study, individual samples are not replicable as they are sampled at discrete time points. Sampling procedure and protocol were consistent throughout the study. Samples were processed in batches, and each batch

	included samples and multiple blanks and mock communities. The blanks and mock communities were served as internal controls to make sure there is no contaminations and instrument bias affecting the final results.
Randomization	Randomization is not relevant to our study because this study is a long-term observation study not a study based on manipulative experiments.
Blinding	Blinding in not relevant to our study, as this study is part of a long term microbial observation project, which has operated for over 20 years. In addition, the sequencing facility that provided the analysis did not know what the sample names correspond to.

Did the study involve field work? [x] Yes [ ] No

Field work, collection and transport

Field conditions	This sampling location is a seasonal subtropical marine ecosystem with temperature ranging from 14 to 24 degree-C, and field conditions vary monthly. All relevant environmental data are presented in Figure 1, and the raw data are provided as a Source data file. For additional information associated with this sampling location please see: https://dornsife.usc.edu/spot/datasets-summary/
Location	Samples were collected from 5m depth at the San Pedro Ocean Time-series (SPOT) location (33.55N, 118.4W).
Access & import/export	Seawater samples were collected monthly from the sampling location by a SeaBird CTD-General Oceanic Rosette assembly with Go-Flo bottles launched from the research vessel Yellowfin from 2005-2018. No permits are necessary for collecting seawater samples from this location for our use. We have not imported or exported the samples.
Disturbance	NA

Reporting for specific materials, systems and methods

We require information from authors about some types of materials, experimental systems and methods used in many studies. Here, indicate whether each material, system or method listed is relevant to your study. If you are not sure if a list item applies to your research, read the appropriate section before selecting a response.

Materials & experimental systems

n/a	Involved in the study	
[x]	[ ]	Antibodies
[x]	[ ]	Eukaryotic cell lines
[x]	[ ]	Palaeontology and archaeology
[x]	[ ]	Animals and other organisms
[x]	[ ]	Human research participants
[x]	[ ]	Clinical data
[x]	[ ]	Dual use research of concern

Methods

n/a	Involved in the study	
[x]	[ ]	ChIP-seq
[x]	[ ]	Flow cytometry
[x]	[ ]	MRI-based neuroimaging
